# Virulent-MDR-ESBL *E. coli and Klebsiella pneumoniae* report from North Sinai calves diarrhea and in vitro antimicrobial by *Moringa oleifera*

**DOI:** 10.1186/s12917-024-04088-7

**Published:** 2024-06-14

**Authors:** Sahar A. Allam, Sara M. Elnomrosy, Samy M. Mohamed

**Affiliations:** 1 Infectious Disease Unit, Animal and Poultry Health Department, Animal and Poultry Production Division, Desert Research Center, 1 Mataria Museum Street, Cairo, 11753 Egypt; 2 Technology Incubator for Nano Agricultural Application, Desert Research Center, 1 Mataria Museum Street, Cairo, 11753 Egypt; 3 Genome Research Unit, Animal Health Research Institute, Agriculture Research Center, Giza, Egypt; 4 Medicinal and Aromatic Plants Research Department, Pharmaceutical and Drug Industries Research Institute, National Research center, Al-Buhouth Street, Dokki, Giza, Egypt

**Keywords:** Virulent genes, ESBL, MDR, *E. coli*, *Klebsiella pneumoniae*, *Moringa oleifera*, Bovine diarrhea, Zoonotic, In silico ferulic acid, Tyrosinase, Egypt

## Abstract

The health of calves has a significant impact on the production of cows and livestock. Some desert plants have pharmacological importance, as they can be used to reduce antibiotic resistance. Our hypothesis is designed to detect Virulent- Multidrug-Resistant and Extended- spectrum Beta- lactamase *Enterobacteriaceae* (Virulent-MDR-ESBL *Enterobacteriaceae* and to determine whether *Moringa oleifera* has antibacterial activity against the detected isolates. A total of 39 *Enterobacteriaceae* isolates from 28 diarrheic samples were collected from calves aged between 20 days and 20 months from 3 different flocks in North Sinai, Sahl-Eltina region, Egypt. *E.coli* 46% (18/39), O157 13% (5/39), *Klebsiella pneumoniae* 41% (16/39). MDR members accounted for 87%, while ESBL isolates accounted for 43%. The antibacterial activity is represented by microdilution. Minimum inhibition concentration (MIC) for the methanol extract of *Moringa oleifera* ranged from 2.5,5,10, and 25mg/ ml among *E.coli* isolates, and O157 was susceptible to (2.5mg/ ml), *Klebsiella pneumoniae* isolates were susceptible to (5-50mg/ ml). Analysis of the methanol extract revealed that ferulic acid was the dominant phenolic compound with a concentration of 29,832 parts per million (ppm). In silico docking study expected the active site of ferulic acid to act on the tyrosine bacterial enzyme through Pi-alkyl, Pi-anion, Carbon hydrogen bonds, and extra ionic attractive interactions with copper ions which can stabilize ferulic acid inside the targeted pocket Diverse virulent gene profiles were observed in *E. coli*. The Shiga toxin-producing *Escherichia coli* (STEC) was reported in 83% of the isolated *E. coli*, while the DNA gyrase (gyrA) was harbored in 100% of *Klebsiella pneumoniae* isolates. Various profiles of antibiotic resistance genes for both *E. coli* and *Klebsiella pneumoniae* isolates were distinguished. bla^TEM^ genes were detected in 99% of *E. coli* and 100% of *Klebsiella pneumoniae*. Sequence analysis for *E. coli* strain DRC-North Sinai-Eg was placed in accession numbers (OP955786) for the Shiga toxin 2 gene (Stx2A), (OP997748) and (OP997749) for the Adhesion to host cell gene (Eae). For the hemolysine gene (hylA), the accession number was (OP946183). *Klebsiella* *pneumoniae* strain DRC-North Sinai-Eg was placed in (OP946180) for (gyrA). This study has proven the broad range of *Moringa oliefera's* antibacterial effects in vitro against the virulent-MDR- ESBL *E. coli* and *Klebsiella pneumoniae* isolated from North Sinai calves diarrhea. These are congruent with the disability effect on bacterial tyrosinase enzyme through docking study therefore, we recommend the usage of this desert plant as a prospective feed additive, we endorse this as an antibacterial new insight natural source and for the medication of considered pathogens with zoonotic impacts.

**Graphical Abstract:**

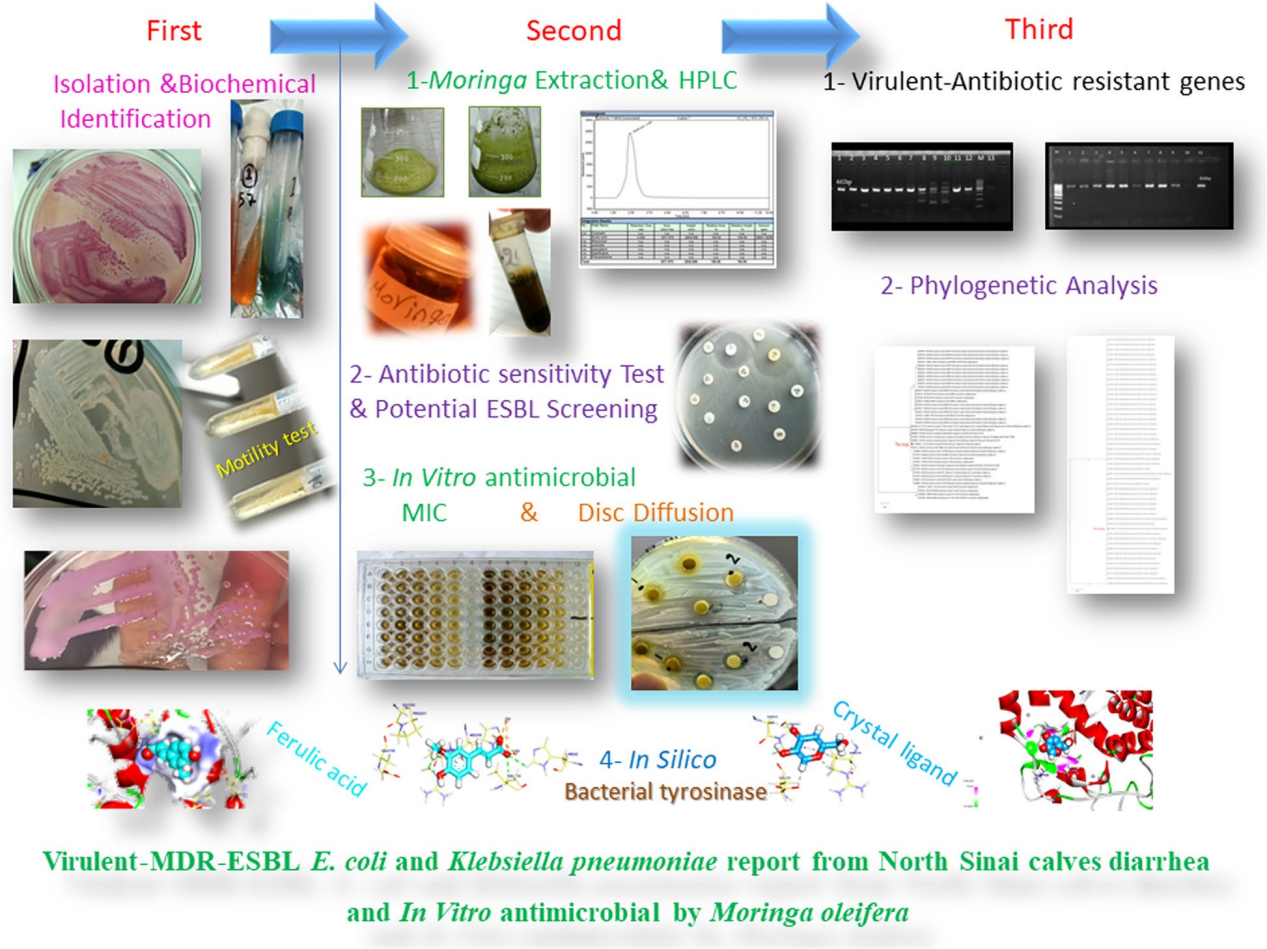

**Supplementary Information:**

The online version contains supplementary material available at 10.1186/s12917-024-04088-7.

## Introduction

Cattle and water buffaloes (Bubalus bubalis) are crucial sources of red meat, milk, and milk products in most developing countries, including Egypt. Newborn calves of this species have a poor immune system, which makes them susceptible to viral and bacterial infections. One of the most common causes of disease and deaths in calves is neonatal calf diarrhea (NCD).

In the dairy industry, approximately 50% of deaths in one-month-old calves are due to diarrhea caused by bacterial, viral, or parasitic pathogens. Viral, bacterial, and protozoan infections cause bovine neonatal diarrhea. As a result, financial harm is due to high mortality, therapeutic disappointments, and weight loss [1].

The eae A gene of enteropathogenic *Escherichia coli* (EPEC) is necessary for intimate attachment to epithelial cells in vitro. Enterohemorrhagic *E. coli *(EHEC) strains also possess an eae gene and are capable of intimate attachment and microvillus effacement in vitro and animal models [2]. Heat-stable toxin (STa) produced by enterotoxigenic *Escherichia coli* (ETEC) [3].

Shiga toxin-producing *Escherichia coli* (STEC) strains have been associated with occurrences of diarrhea, hemorrhagic colitis, and hemolytic-uremic syndrome in humans. A result of the production of Shiga toxins 1–2 (Stx 1–2), or combinations of these toxins represent the most clinical signs of infection. Other main virulence factors contain enterohemorrhagic *E. coli* hemolysin (EHEC hlyA) and intimin, the creation of the eaeA gene that is included in the attaching and adherence phenotype. STEC isolates definite virulence in humans. These strains are often referred to as verotoxin-producing *E. coli* due to the effect these Stx 1–2 require on Vero cells in culture. These toxins affect not only animals but also humans. The global cause of severe human gastrointestinal disease is a trait of toxigenic isolates, often through bloody diarrhea, hemorrhagic colitis (HC), and hemolytic-uremic syndrome [4].

In recent years, the pharmaceutical industry has focused on the discovery of new bioactive compounds as antiviral agents, and the main focus in recent decades for pharmaceutical discovery from natural products has been on microbial sources (bacterial and fungal), dating back to the discovery of penicillin from the mold fungus *Penicillium notatum* in the first half of the twentieth century. The first investigation of the antibiotic activity of algae was carried out by (Pratt et al. 1944) [5].

The idea of plants to secrete secondary metabolites is a defense mechanism against pathogen infections. These metabolites are toxic and contain mainly secondary metabolites [6].

*Moringa oleifera* *Lam.* is a family *Moringaceae* tree known as resident in numerous tropical and subtropical countries and widely used in traditional medicine due to its leaves, seeds, bark, roots, sap, and flowers [7].

The World Health Organization (WHO) reported more than 80% of the world’s population relies on traditional medicines for their most important healthcare needs. The most important bioactive compounds of plants are alkaloids, flavonoids, tannins, and phenolic compounds. It has been reported for its role in food preservation due to its antibacterial activity [8].

Antibiotics are commonly used to treat bovine neonatal diarrhea, but extreme application resulted in adverse effects on human and animal health, as well as direct dangerous effects on the environment [9].

The objective of the current study is to assess the phenotypic and genotypic of virulent and resistance genes of some antimicrobial drugs in *Enterobacteriaceae* that had been isolated from diarrheic calves in North Sinai. Subsequence in vitro testing of *Moringa oleifera* as a natural desert plant rich source of phenolic compounds that might serve against virulent and multidrug resistance which affect the cow production^,^ s health or cause zoonotic impact. In silico bioinformatics of the dominant component of *Moringa oliefera* extract to support the in vitro results. Therefore, this study might solve that problem as prospective animal feed additive.

## Material and methods

This study was performed on 28 calf residents in North Sinai, Sahl-Eltina region, Egypt. Animals under study were privately owned by individuals, we asked them for diarrheic samples from their calves during desert research center coverage’s veterinary services for this region within the intervals between 2020–2021. The ages of these calves ranged between 10, 12, 18, 20 months, and 20 days. The samples were collected starting from November 2020.

### Sampling, isolation, and identification procedures

Fecal swabs were prepared in MacConkey's broths and incubated overnight at 37 °C. Each sample was inoculated on MacConkey agar, Biolife (Viale Monza, 272- 20,128 Milano). O157 isolates were confirmed on MacConkey agar with sorbitol Biolife (Viale Monza, 272- 20,128 Milano). Mucoid appearance was tested on MacConkey agar, Hektoen agar and MacConkey agar with sorbitol also. Motility test was done to differentiate *Klebsiella pneumoniae* with other mucoid *Enterobacteriaceae.* Biochemical tests (Triple sugar Iron (TSI), urease, citrate, Methyl red, Voges Proskauer (VP) test and Indole test were performed to differentiate between *E. coli* and Klebsiella pneumonae [10, 11].

### Antibiotic sensitivity test

A different 11 antibiotics [12] were listed in Supplement file, Table 1S and were used in antibiotic sensitivity tests [13]. The parameters of multidrug resistance [14] considered non-susceptibility to three or more antibiotics of different groups. Test was performed on Mueller Hinton agar (Biolife Italiana srl).

**Table 1:** Primer names, target genes, oligonucleotide sequences, and the product sizes used in PCR for *E. coli* virulence genes

Primer target	Primer name	Sequence of primers(5’-3’)	Annealing temperature (^0^C)	Product size	Reference
Shiga toxin1	Stx1F	ATAAATCGCCATTCGTTGACTAC	60	180 pb	[15, 16]
	Stx1R	AGAACGCCCACTGAGATCATC	60		
Shiga Toxin 2	Stx2F	GGCACTGTCTGAAACTGCTCC	60	254 pb	
	Stx2R	TCGCCAGTTATCTGACATTCTG	60		
Hemolysine enzyme	hyl A F	GCATCATCAAGCGTACGTTCC	60	534 pb	
	hylA R	AATGAGCCAAGCTGGTTAAGCT	60		
Adhesion to host cell	Eae F	GACCCGGCACAAGCATAAGC	60	384 pb	
	Eae R	CCACCTGCAGCAACAAGAGG	60		
Shiga‐like toxin I	SLTI-5	AGCTGAAGCTTTACGTTTTCGG	60	590 pb	
	SLTI-3	TTTGCGCACTGAGAAGAAGAGA	60		
Shiga‐like toxin II	SLTII-5	TTTCCATGACAACGGACAGCAGTT	60	694 pb	
	SLTII-3	ATCCTCATTATACTTGGAAAACTCA	60		

### Potential ESBL-producing screening *Enterobacteriaceae* isolates

According to the Clinical & Laboratory Standards Institute [17], screening was performed for the probability presence of ESBL among the obtained isolates. The isolates that displayed inhibition zones with third generation cephalosporine (ceftazidime (30microgram (µg) and/or cefotaxime (30 µg) of 22 mm and/or 27 mm) were regarded as potential ESBL producers (ESBL-positive screening).

### *Moringa oleifera* preparation

*Moringa* leaves were obtained by Professor Doctor Samy Mohamed, Medicinal and Aromatic Plants Research Department, Pharmaceutical and Drug Industries Research, National Research Center, Al-Buhouth Street, Dokki, Giza, Egypt.

### *Moringa* methanol extraction

About 20–30 grams of fresh leaves were washed with water, it was dried, and followed by boiling with 200 milliliter (mL) of solvent for 1hour (h). Filtration was performed by Whatman filter paper No. 1 and then concentrated in a vacuum at 40°C-50°C using a rotary evaporator. Evaporation of the solvent in the rotary evaporator yielded a crude extract of the soluble components. High-performance liquid chromatography (HPLC) was applied to the extract [18].

### High-Performance Liquid Chromatography (HPLC)

HPLC analyses of *Moringa oleifera* extract were done in the Microanalysis Unit Laboratory Complex at the Desert Research Center according to (Biswas et al. 2013) [19]. Briefly, the Thermo (Ultimate 3000) system was made up of a pump, an automatic sample injector, and a DELL-compatible computer that was connected to it and supported the Cromelion7 interpretation program. A DAD-3000 diode array detector was employed. Operating at 25°C, a Thermohypersil reversed phase C18 column 2.5 30 cm. Trifluoroacetic acid or acetonitrile (0.05%, solvent A) and distilled water (solvent B) were used as the mobile phase. The Ultra Violet (UV) absorption spectra of the samples and the standards were captured between 230 and 400 nanometers (nm). Degassing was achieved before samples, standard solutions, and the mobile phase were filtered through a 0.45 millipore (m) membrane filter. By comparing the compounds' UV absorption spectra and retention times to those of the standards, the compounds were identified.

### In vitro inhibitory effects of *Moringa oleifera*

#### Minimum Inhibition Concentration (MIC) microtiter plate method

The antibacterial activity was determined by MIC microtiter plate methods [20–23]. McFarland turbidity standard to adjust densities of bacterial suspension 24 h growth was prepared. Preparation of 1milligram/milliliter (mg/ml) stock extract. Dimethylsulfoxide (DMSO) was serially diluted in a 96-well microtiter plate and inoculated as follows; 100µl nutrient broth was filled in all wells, and Lane 1 received 100µl of the first extract concentration. Two fold dilutions were accomplished resulting in concentrations of 1000, 500, 250, 125, 62.5, 31.25, 15.6, 7.8, and 3.9 µg/100 µl (µg/100 µl). 90µl of nutrient broth and 10µl of freshly prepared isolate broth. Four controls were regarded in each run, the serially diluted extract of each concentration was kept without bacterial inoculation, one well of 100µL DMSO was mixed with the isolate suspension, one well for each isolate without inoculation, and amikacin 30µg/100µl. Incubation was performed at 37°C. Finally, the samples were read with an Enzyme Linked Immunosorbent Assay (ELISA) reader at 630 nm. (Biotek Instrument).

#### Minimum Bactericidal Concentration (MBC)

MBC was determined [22]. Five microliters from each detected MIC were cultured on Mueller Hinton agar (Biolife Italiana srl) under aseptic conditions and incubated at 37 °C, the MIC where no growth was determined (MBC).

#### Disc diffusion

The antimicrobial effect confirmation was achieved by the disc diffusion assay. Approximately 5 mm sterile discs were saturated with 30 µl of 30 to 100 µg extract. Twenty microliters of freshly prepared samples of each *Enterobacteriaceae* isolate were streaked over Mueller Hinton agar (Biolife Italiana srl) by using a sterile loop. A saturated DMSO disc was used as a control.

###  In silico inhibitory effects of ferulic acid docking study

Ferulic acid was docked by using MOE 19.0901 Software to examine its binding to the bacterial tyrosinase enzyme, Crystal proteins; the protein Data code (PDB code: 3NQ1; https://www.rcsb.org) have binding sites created by the cocrystalized ligand. Molecules of water were not included. After that, the protein was quickly prepared, missing amino acids were added, and empty valence atoms were filled in. The Merck molecular force field (MMFF94) was used to reduce the energy. Ferulic acid was allowed to move freely, and the proteins were rigid because docking was accomplished using the flexible molecule technique.

### Characterization of virulence genes and antibiotic resistance genes for *Enterobacteriaceae* isolates

#### Deoxyribonucleic acid (DNA) extraction

DNA extraction was completed using a QIAamp® DNA Mini Kit (Qiagen, Germantown, USA). Nucleic acid was eluted with 100 µl of elution buffer provided in the kit. A nanodrop spectrophotometer was used to measure the concentration and purity at an optical density of 260/280.

### Polymerase Chain Reaction (PCR)

The AmpliTaq Gold® Fast Master Mix (www.lifetechnologies.com); 2 µl Forward primer (F); 2 µl Reverse primer (R); 3.5 µl water; and 5 µl DNA made up the entire 25 µl volume of the reaction mixture. Following is the heat profile for 40 cycles: initial denaturation at 95 °C for 10 min; denaturation at 95 °C for 3 s; annealing at the temperature listed in the primers' table for 3 s; and extension at 68 °C for 5 s, 15 s of final extension at 72 °C. Primer sequences, [15, 16, 24, 25] target genes and PCR products are summarized in Tables 1 and 2. Antibiotic resistance gene primers [26] were placed in Table 3. The PCR products were loaded on 1.5% agarose gel (Applichem, Germany, GmbH) that was stained with ethidium bromide. The amplified DNAs were electrophoresed at 5 Volt/centimeter (V / cm) for 30 min on a mini horizontal electrophoresis unit. A 100 bp DNA Ladder (Qiagen, Germany, GmbH) was used to determine the fragment sizes. The gel was then visualized and photographed under an ultraviolet transilluminator.

**Table 2:** Primer names, target genes, oligonucleotide sequences, and the product sizes used in PCR for *Klebsiella pneumoniae*

Primer target	Primer name	Sequence of primers(5’-3’)	Product size	Reference
Encoding the DNA gyrase A subunit in *Klebsiella pneumoniae*	gyrA -F	CGCGTACTATACGCCATGAACGTA	441 pb	[25]
	gyrA -R	ACCGTTGATCACTTCGGTCAGG

**Table 3:** Primers used for antibiotic resistance genes

Primer target	Primer name	Sequence of primers(5’-3’)	Annealing temperature (^0^C)	Product size (bp)	Reference
Beta-lactamases enzymes causing ESBL	TEM-F	ATG AGT ATT CAA CAT TTC CGT	58	861	[26]
	TEM-R	TTA CCA ATG CTT AAT CAG TGA			
	SHV-F	CGC CTG TGT ATT ATC TCC CTG	64	849	
	SHV-R	TTA GCG TTG CCA GTG CTC GAT			
	CTX-M 9 group-F	GCG TGC ATT CCG CTG CTG C	67	832	
	CTX-M 9 group-R	ACA GCC CTT CGG CGA TGA TTC			

### Sequence and phylogenetic characterization

Using the QIA quick Gel Extraction Kit (Qiagen, Hilden, Germany), as directed by the manufacturer, DNA bands of the anticipated size were removed from the gel and purified. ABI PRISM Big Dye Terminators v3.1 Cycle Sequencing Kits were used to directly sequence the purified PCR results (Applied Biosystems, Waltham, Massachusetts, USA). A Centrisep purification kit was used to clean up the sequencing reaction products (Applied Biosystems). An ABI PRISM3500 genetic analyzer was used to sequence the purified products directly (Applied Biosystems). Sequence identities were initially verified using Basic Local Alignment Search Tool (BLAST)® (https://blast.ncbi.nlm.nih.gov/Blast.cgi) analysis. The evolutionary history was inferred by using the Maximum Likelihood and Tamura Nei Model [27] Mega11 was used for evolutionary analysis [28] (Tamura et al., 2021).

### Statistical analysis

Statistical analysis (Microsoft Excel) was tested by Analysis Of Variance (ANOVA) single factor, Probability Value (*P* value < 0.05) between mean values of antibiotic Susceptibility (S), Intermediate (I), and Resistance (R) percentages among *E. coli* and *Klebsiella pneumoniae* isolates, respectively, and between the mean values of *Moringa* *oleifera* MIC against all studied isolates.

## Results

### Isolation

**Fig. 1:**
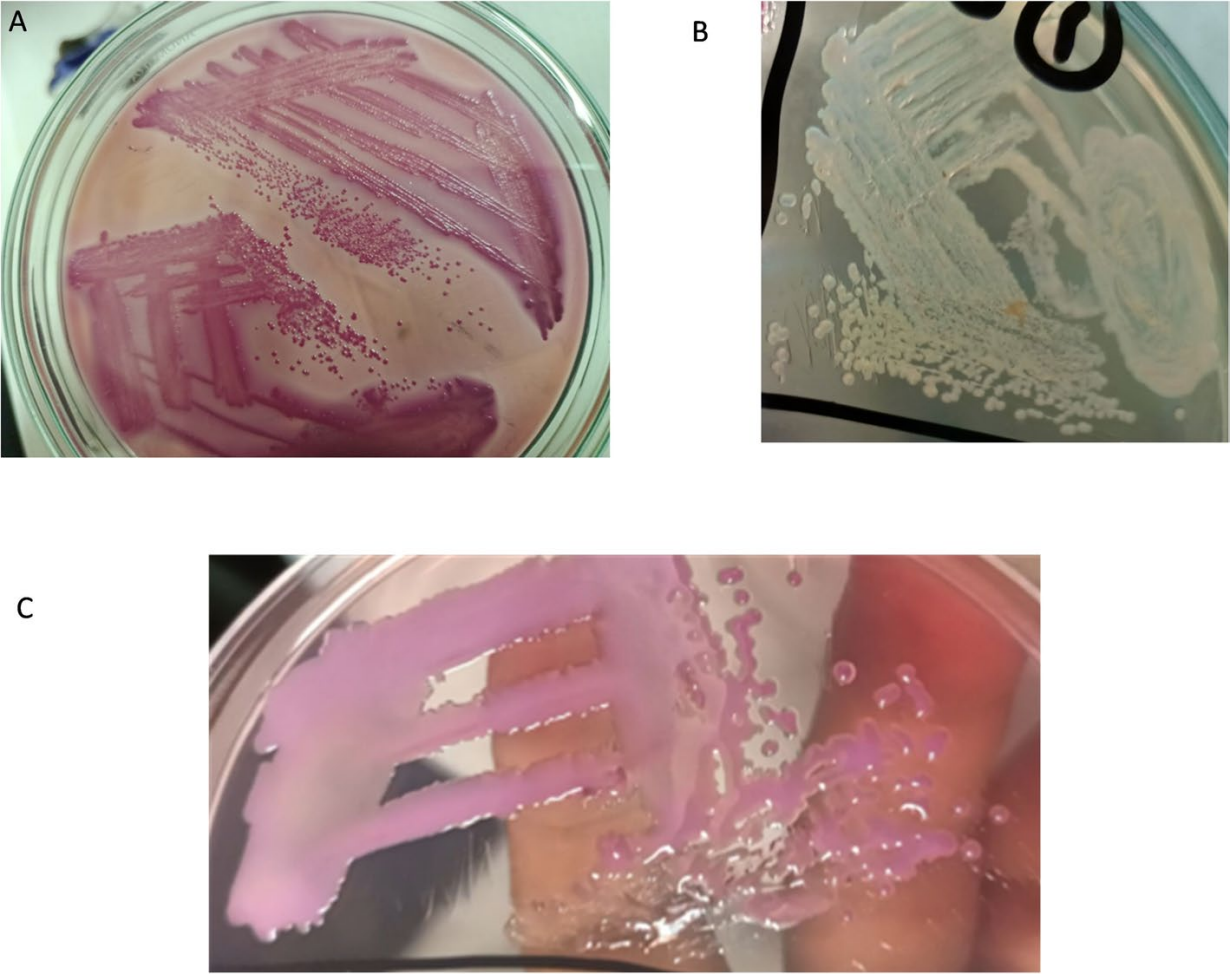
Characterization of *Enterobacteriaceae* isolated from diarrheic calves on MacConkey agar with sorbitol. **A** sorbitol fermenter pink colonies *E. coli*, **B** non-sorbitol fermenter pale white colonies of O157. **C** Mucoid colonies of *Klebsiella pneumonia*e

As shown in Table 4, A total of 39 *Enterobacteriaceae* were isolated from 28 calves among three different flocks. Well-known non-sorbitol fermenter O157 colonies on MacConkey agar with sorbitol. Lactose and sorbitol fermenter pink colonies of *E. coli* and highly mucoid appearance of *Klebsiella pneumoniae* on both MacConkey agar and MacConkey agar with sorbitol, respectively. Non-motile *Klebsiella pneumoniae* differentiated from other mucoid *Enterobacteriaceae* Fig. 1.

**Table 4:** Types and frequency of *Enterobacteriaceae* isolated from diarrheic calves in North Sinai

^a^*N* ^o^ of flocks	*N* ^o^ of calves	Age	*E. coli*	O157	*Klebsiella pneumoniae*
1st flock	1	10 ^b^d	+	+	-
	2	11d	-	+	+
	3	7d	+	+	+
	4	12d	+	-	-
	5	10d	+	-	+
	6	8d	+	-	+
	7	12d	+	-	-
	8	15d	+	-	+
	9	13d	-	-	-
	10	10d	-	-	-
	11	10d	-	-	-
	12	11d	+	-	+
	13	18d	-	-	-
2nd Flock	1	18d	-	+	-
	2	10d	-	-	+ + Variant
	3	10d	+	-	+
	4	10d	-	-	-
	5	10 d	+	-	+
	6	12 d	+	+	+
	7	1 ^c^M	+	-	-
	8	5 M	+ + Variant	-	+ + Variant
	9	20 d	+	-	+
3rd Flock	1	15d	-	-	-
	2	18d	-	-	-
	3	16d	+	-	-
	4	15d	-	-	-
	5	20d	+	-	+
	6	18d	+	-	+
Total	28		18	5	16
Frequency per 39 isolates			46% (18/39)	13% (5/39)	41% (16/39)

^a^*N*^o^ Numbers, ^b^d days, ^c^M Months

Biochemical test confirmation for both *E. coli* and *Klebsiella pneumoniae* isolates were assessed that fermentation of glucose in TSI test revealed acid slant and butt with gas evolved without H_2_S, positive sorbitol and lactose fermentation. *E. coli* isolates were negative urease, negative citrate, positive Methyl red, negative VP test, and positive indole test. *Klebsiella pneumoniae* isolates were positive citrate, positive urease, indole test was negative, and methyl red was negative, positive VP test. Confirmation was performed that *E. coli* were 46% (18/39), O157 were 13% (5/39), and *Klebsiella pneumoniae* were 41% (16/39).

### Antibiotic sensitivity test

An antibiotic sensitivity test was carried out on 30 selected field isolates. Erythromycin and azithromycin resistance was 86% and 79%, respectively, and amoxicillin and gentamycin resistance was 57%. O157 isolates were fully resistant (100%) to gentamycin. Complete resistance of *Klebsiella pneumoniae* to erythromycin, azithromycin, and amoxicillin (Table 5).

**Table 5:** Antibiotic sensitivity test on *E. coli and Klebsiella pneumoniae* isolated from diarrheic calves

Antibiotics ^a^(mm) Isolates	MDR, or NO	F (300)	LE (5)	CN (10)	AK (30)	E (15)	CFR (30)	AZM (15)	DO 30	AX (25)	**CAZ** **(30)**	NOR (10)
**MDR-*E.coli*** 78.6% (11/14)	MDR-3	20S	36S	8R	22S	14I	19S	10R	20S	R	26S	24S
	MDR-3	24S	24S	15I	23S	R	19S	R	15S	R	24S	20S
	MDR-4	24S	18I	16I	23S	11R	16S	R	20S	R	28S	R
	NO	23S	32S	14R	24S	19I	18S	14S	24S	14I	23S	26S
	MDR-5	22S	40S	15I	23S	R	14R	10R	10R	R	25S	28S
	MDR-9	22S	R	R	20S	R	R	R	R	R	R®	R
	MDR-3	26S	28S	12R	23S	11R	15S	16S	R	30S	25S	26S
	NO	20S	38S	15I	22S	11R	16S	7R	24S	23S	26S	27S
	MDR-3	26S	32S	13R	24S	R	18S	8R	28S	22S	26S	28S
	MDR-5	24S	25S	11R	25S	R	15S	R	12I	R	22 ESBL®	14I
	NO	26S	28S	17S	22S	11R	15S	10R	18S	17I	26S	25S
	**MDR-3**	**25S**	37S	19S	26S	9R	20S	R	22S	10R	26S	26S
	MDR-4	26S	38S	13R	26S	11R	12R	8R	28S	14I	28S	28S
	MDR–3	24S	40S	8R	24S	R	22S	14S	30S	8R	25S	20S
***E. coli*** ** Resistance%**		**NO**	**7% 1/14**	**57%** **8/14**	**NO**	**86%** **12/14**	**21% 3/14**	**79%** **11/14**	**21%** **3/14**	**57%** **8/14**	**14%** **2/14**	**14%** **2/14**
**MDR O157** **67%(2/3)**	NO	30S	40S	4R	26S	14I	17S	14S	10R	19S	29S	32S
	MDR-4	26S	34S	12R	13R	10R	20S	R	11I	21S	25S	25S
	MDR-7	20S	22S	R	11R	R	12R	R	8R	18S	18®	17I
**O157 Resistance%**		NO	NO	100% 3/3	67% 2/3	67% 2/3	33% 1/3	67% 2/3	66.7% 2/3	NO	33% 1/3	NO
**MDR-** ***Klebsiella pneumoniae*** **100%(13/13)**	MDR-4	22S	42S	19S	32S	R	22S	11R	8R	R	26S	24S
	MDR-7	24S	18I	16I	24S	12R	R	11R	12R	R	R®	R
	MDR-7	R	23S	15I	28S	R	R	R	13I	R	7®	12R
	MDR-8	25S	24S	12R	20S	13R	R	R	R	R	R®	12R
	MDR-8	14R	30S	14R	20S	R	13R	R	24S	R	22® ESBL	21S
	MDR-7	R	28S	14R	26S	R	R	R	14S	R	R®	22S
	MDR-8	R	28S	14R	20S	R	R	R	8R	R	R®	24S
	MDR-5	15I	18S	15S	20S	12R	R	R	21S	R	20®	24S
	MDR-6	22S	28S	13R	23S	10R	10R	R	12S	R	R®	14I
	MDR-8	R	28S	12R	10R	R	R	R	24S	R	R®	20S
	MDR-8	14R	14R	14R	23S	R	14R	R	24S	12R	27S	R
	MDR-4	24S	38S	12R	23S	12R	16I	11R	15S	R	25S	28S
	MDR-5	37S	34S	18S	24S	R	R	R	16S	R	R®	24S
***Klebsiella pneumoniae*** ** Resistance%**		46% 6/13	7.7% 1/13	62 8/13	7.7% 1/13	100%13/13	85% 11/13	100% 13/13	31% 4/13	100% 13/13	77% 10/13	31% 4/13

^a^mm (millimeter), ®; Positive extended spectrum Beta-lactamase (ESBL) ≤ 22 mm

F (Nitrofurantoin 300 Micrograms/disc (mcg/Disc). S** ≥** 17 I = (15–16), R ≤ 14 mm LE (Levofloxacine 5mcg/Disc)S ≥ 21,I = (17–20), R ≤ 16 mm

CN (gentamycin 10 mcg/disc)S ≥ 15, I = (13–14), R ≤ 12 mm AK (amikacin (30 mcg/disc) S ≥ 17, I = (15–16), R ≤ 14 mm

E (Erythromycin 15 mcg/Disc) S ≥ 23, I = 14–22, R ≤ 13 mm CFR (Cefadroxil 30 mcg/Disc) S ≥ 15, (I(Non), R ≤ 14 mm

AZM (azithromycin 15 mcg/disc) S ≥ 13 I(non), R ≤ 12 mm CAZ (ceftazidime 30 mcg/disc) S ≥ 21, I = (18–20), R ≤ 17 mm

DO (Doxycycline 30 mcg/Disc S ≥ 14, I = (11–13)), R ≤ 10 mm NOR (Norfloxacin-Fluoroquinolone 10 mcg/Disc) S ≥ 17, I = (13–16), R ≤ 12 mm

AX (Amoxicillin 25 mcg/Disc)S ≥ 18, I = (14–17), R ≤ 13 mm

ESBL was observed in 43% (13/30) of isolates among the selected tested *Enterobacteriaceae*, as *E. coli* isolates 14% (2/14) were ESBL, O157 represented 33% (1/3) and *Klebsiella pneumoniae* represented 77% (10/13). Multidrug resistance among *Enterobacteriaceae* was detected in 87% (26/30) as follows:* E. coli* isolates was 78.6% (11/14), O157 represented 67% (2/3), while it was 100% (13/13) among *Klebsiella pneumoniae*.

ANOVA single factor revealed statistically significant differences (*P* value < 0.05) between mean values of Susceptibility (S), Intermediate (I), and Resistance (R) percentages in *E. coli* (*P* value = 0.006963189) and *Klebsiella pneumoniae* (*P* value = 0.026583233) Table 6.

**Table 6:** ANOVA-single factor to antibiotic susceptibility test among the isolated *Enterobacteriaceae*

Antibiotics	^*^ *E. coli* (14)			^*^O175 (3)			^*^Klebsiella pneumoniae (13)		
	S	I	R	S	I	R	S	I	R
Nitrofurantoin	14(100%)	NO	NO	3(100%)	NO	NO	6(46%)	1(7.7%)	6(46%)
Levofloxacin	12(85%)	1(7.1%)	3(7%)	3(100%)	NO	NO	11(85%)	1(7.7%)	1(7.7%)
Gentamycin	2(14.3)	4(28.6%)	10(57%)	NO	NO	3(100%)	3(23%)	2(15.4%)	8(62%)
Amikacin	14(100%)	NO	NO	1(33%)	NO	2(67%)	12(92.3)	NO	1(7.7%)
Erythromycin	NO	2(14.2%)	12(86%)	NO	1(33%)	2(67%)	NO	NO	13(100%)
Cefadroxil	11(78.6%)	NO	3(21%)	2(67%)	NO	1(33%)	1(7.7%)	1(15.4%)	**11(85%)**
Azithromycin	3(21.4%)	NO	11(79%)	1(33%)	NO	2(67%)	NO	NO	13(100%)
Doxycycline	10(71.4%)	1(7.1%)	3(21%)	NO	1(33%)	2(67%)	8(62%)	1(15.4%	4(31%)
Amoxicillin	3(21.4%)	3(21.4%)	**11(57%)**	3(100%)	NO	NO	NO	NO	**13(100%)**
Ceftazidime	12(85.7%)	NO	2(14%)	2(67%)	NO	1(33%)	3(23%)	NO	**10(78%)**
Norfloxacin-Fluoroquinolone	11(78.57%)	1(7.1%)	2(14%)	2(67%)	1(33%)	NO	8(62%)	1(15.4%)	4(31%)
F	6.278300953			2.710663174			4.297137067		
*P* value	^*^0.006963189			^*^0.098867795			^*^0.026583233		
F crit	3.443356779			3.682320344			3.443356779		

^*^ANOVA: Single Factor. F > F crit and *P* value < 0.05, so there were statistically significant differences^a*^ between mean values of antibiotic susceptibility, intermediate and resistance percentage

### In vitro inhibitory effects of *Moringa oleifera*

#### MIC, MIG, and Disc Diffusion

Antibacterial activity tested for the methanol extract of *Moringa oleifera* on some selected isolates, as shown in Table 7**.** *Moringa oleifera* had MIC ranges of 5–12.5mg/ ml against 13 isolates of *E. coli* and 10–50mg/ ml for MIG. However, O157’s three isolates had an MIC of 2.5mg/ ml, but their MIG was more than 50mg/ ml. Nine selected *Klebsiella pneumoniae* isolates had a range of 5–50mg/ ml and were equal to or more than 50mg/ ml*.* Disc diffusion performed for visual confirmation of antibacterial activities*.* Sensitivity observed at concentrations between 30 and 60 mg/ ml DMSO (disc loaded with 20ul). ANOVA single factors showed statistically significant differences between mean values of MIC, that is, F > F critical (F crit) and *P* value = (0.047527396) < 0.05.

**Table 7:** Minimum inhibition concentration (MIC) and minimum inhibition growth (MIG) for some selected *Enterobacteriaceae* isolates

Test Isolates (N^o^)	^*^MIC mg/ ml	MIG mg/ ml
*E. coli* (13 isolates)	12.5	50
	10	20
	10	50
	25	50
	10	50
	10	50
	10–2.5	> 50
	10–20	> 50
	5	> 50
	5	= 5
	5	10
	6.5–12.5	10
O157 (3 isolates)	2.5	+ 50
	2.5	
	2.5	
*Klebsiella pneumoniae* (9 isolates)	25	± 50
	5–10	± 50
	25	± 50
	5–20	± 50
	5	± 50
	20–50	± 50
	25	< 50
	6.5–25	< 50
	50	< 50
*F*	3.534425198	
*P value*	^*^0.047527396	
*F crit*	3.466800112	

^*^ANOVA Single Factor. F > F crit and *P* value < 0.05, so there were statistically significant differences^*^ between the *Moringa oliefera* MIC mean values against all tested isolates

#### HPLC

**Fig. 2:**
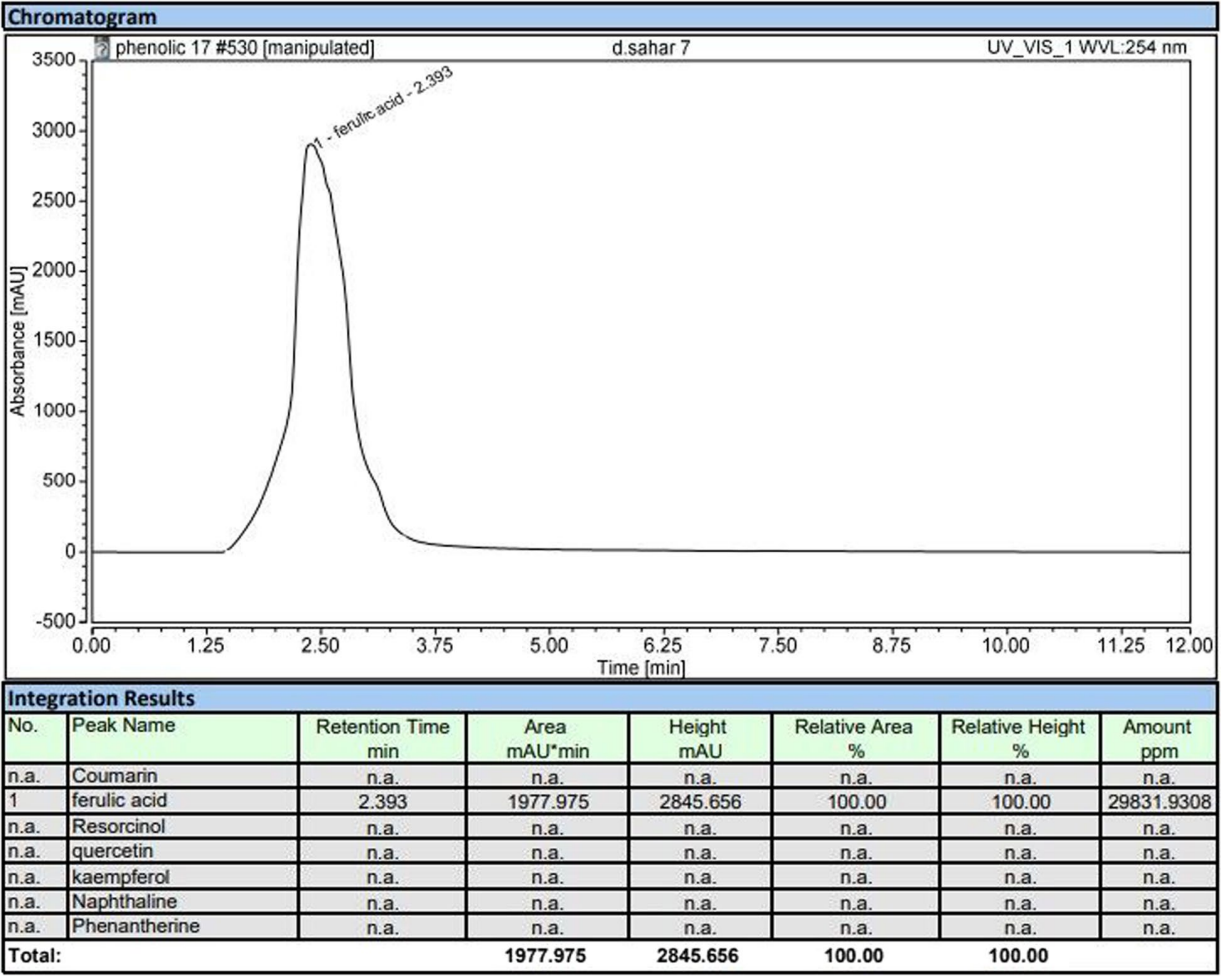
HPLC fingerprint of ferulic acid extract of *Moringa oleifera*

HPLC for the methanol extract revealed that ferulic acid was the dominant phenolic compound with a concentration of 29,832 ppm. As shown in Fig. 2.

###  In silico predicted inhibitory effects of Ferulic acid docking study

**Fig. 3:**
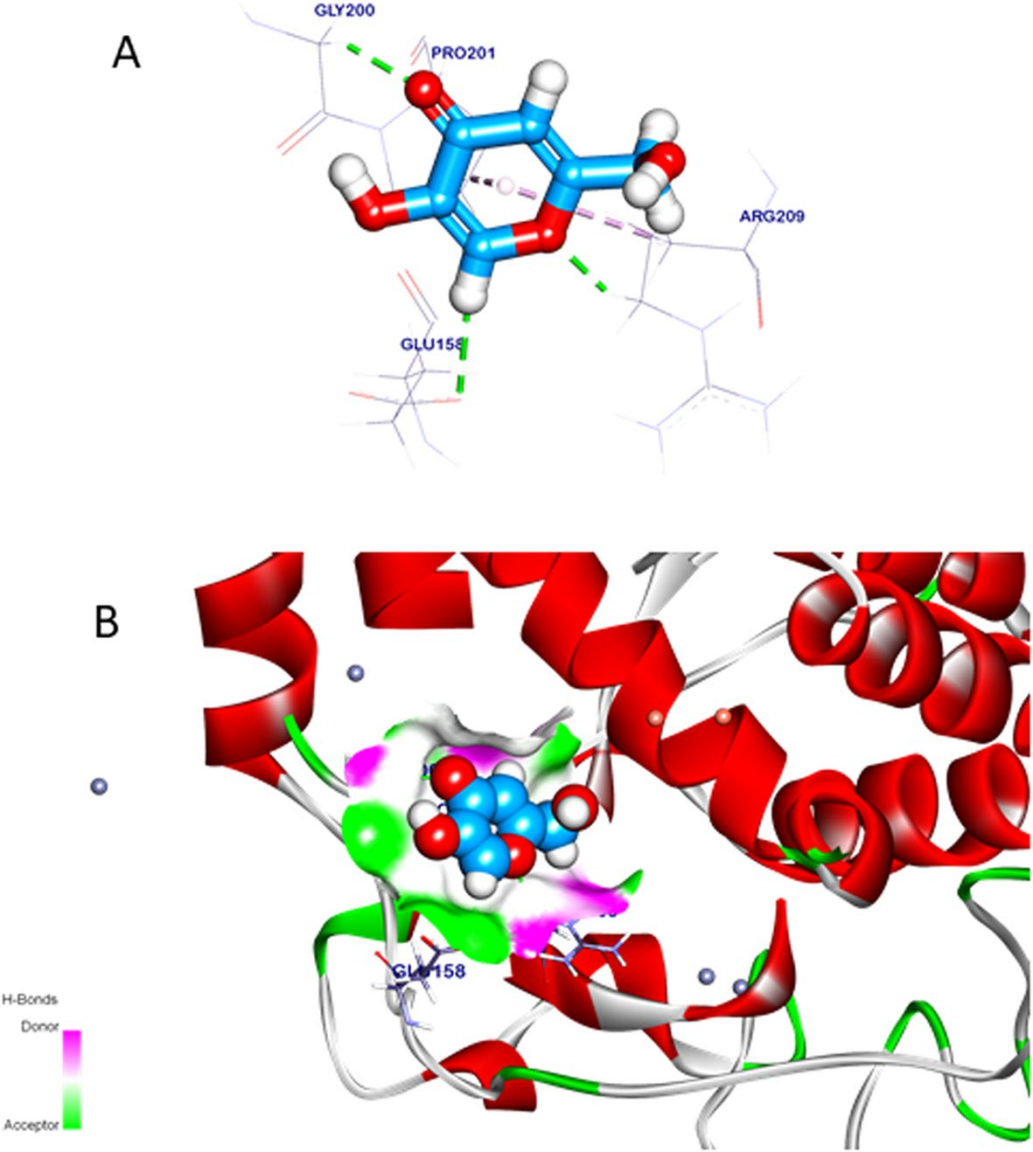
Crystal ligand docked against bacterial tyrosinase, hydrogen interactions are presented with a green line, and the pi interactions are shown in purple lines (**A**) with surface mapping showing crystal ligand occupying the active pocket of bacterial tyrosinase (**B**)

The binding orientation of the crystal ligand exhibited an energy binding of -4.84 kcal/mol against bacterial tyrosinase. The crystal ligand formed two pi-alkyl interactions with Proline (Pro201) and Arginine (Arg209) and additionally interacted with Arg209, Glutamine (Glu158), and Glycine (Gly200) by three carbon-hydrogen bonds (H.B), with distances of 2.40, 2.72, and 2.52 Angstroms (Å). Figure 3 Affinity scores and binding orientations were recorded and were collected in Table 8.

**Table 8:** Binding energy (DG) and root-mean-square deviation (RMSD) of atomic positions interactions kilocalorie/mole (kcal/mole) of tested ligands against targeted sites of Bacterial tyrosinase

Targets screened	Tested compounds	RMSD value (Å)	Docking (Affinity) score (kcal/mole)	Interactions	
				H.B	Pi -interaction
Bacterial tyrosinase	Crystal ligand	1.46	-4.84	3	2
	Ferulic acid	1.22	-5.65	4	2

**Fig. 4:**
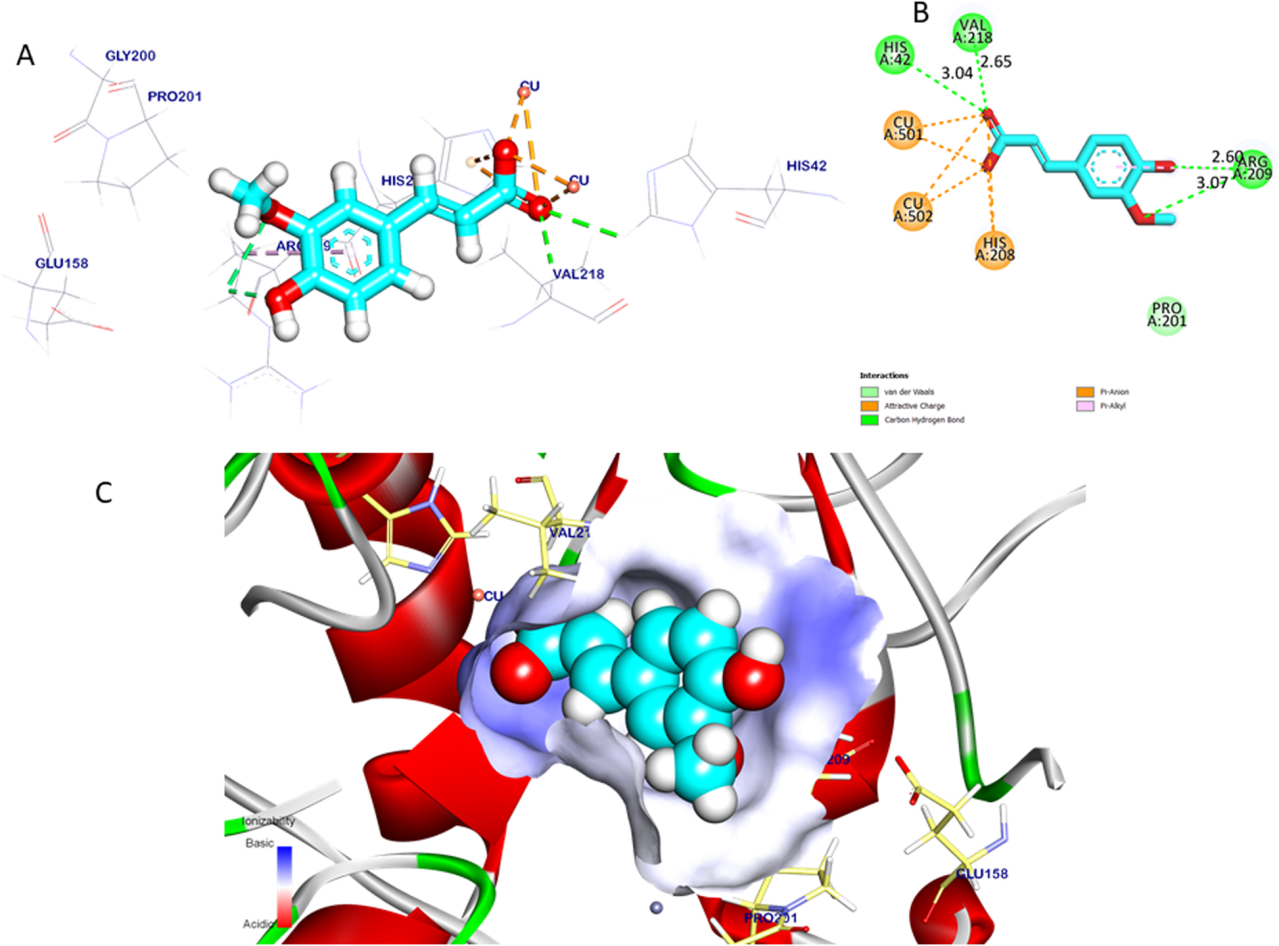
Ferulic acid docked in bacterial tyrosinase, hydrogen interactions are presented with a green line, and the pi interactions are shown with purple lines (**A**), (**B**) with surface mapping showing ferulic acid occupying the active pocket of bacterial tyrosinase (**C**)

The binding interactions of ferulic acid exhibited an affinity binding of -5.65 kcal/mol against bacterial tyrosinase. Ferulic acid formed two Pi-alkyl and Pi-anion interactions with Arg209 and Histidine (His208) and interacted with His42, Valine (Val218), and Arg209 by four carbon-hydrogen bonds with distances of 3.04, 2.65, 2.60, and 3.07 Å. Additionally; ferulic acid interacted with copper ions by ionic attractive interactions, which can stabilize ferulic acid inside the targeted pocket Fig. 4.

### Virulent and antibiotic resistance genes

Different virulent gene profiles were observed in *E. coli* Table 9. The proportion of virulent genes was 83% (10/12) for Stx1, 33% (4/12) for Stx2, 8% (1/12) for Eae, 25% (3/12) for hylA, 33% (4/12) for SLTI, and 33% (4/12) for SLTII. They were frequently found in *E. coli*. As shown in Supplement file, Figs. 1S and 2S. All tested *Klebsiella pneumoniae* isolates had a 100% of *Klebsiella pneumoniae* gyrA genes (13/13). Supplement file Fig. 3S.

**Table 9:** Profiles of *E. coli* virulence gene

Isolates	virulent gene profile
1- O157	Stx2
2- O157	Stx1
3- *E Coli*	Stx1, hylA
4- *E Coli*	Stx1, SLTI
5- *E Coli*	Stx1, Stx2, SLTI, SLTII
6- *E Coli*	Stx1, Stx2, Eae, hylA, SLTII
7- *E Coli*	Stx1, SLTI, SLTII
8- *E Coli*	Stx1, Stx2, SLTII
9- *E Coli*	Stx1
10- *E Coli*	Stx1
11- *E Coli*	SLTI
12- *E Coli*	Stx1, hylA

We selected TEM, SHV, and CTX-M as belonging to the main ESBL types. Antibiotic resistance genes were reported in different patterns in Table 10. bla^TEM^ genes were detected in 99% (11/12), bla^SHV^ in 67% (8/12), and bla ^CTX−M9^ in 8% (1/12) of *E. coli*. On the other hand, bla^TEM^ identified 100% (13/13) *of Klebsiella pneumoniae* isolates*,* Supplement file, Fig.4S, bla ^CTX−M 9^ reported 31% (4/13) of *Klebsiella pneumoniae* isolates, and bla^SHV^ reported none of the tested isolates.

**Table 10:** Profiles of antibiotic resistance genes

*E Coli*		*Klebsiella pneumoniae*	
bla^TEM^, bla ^SHV^	66.7%(8/12) (O157 were included)	bla^TEM^	77%(10/13)
bla^TEM^	16.6%(2/12)	bla^TEM^, bla^CTX−M 9^	31%(4/13)
bla^TEM^, bla^CTX−M 9^	8.3%(1/12)	bla ^SHV^	NOT detected

### Sequencing

**Fig. 5:**
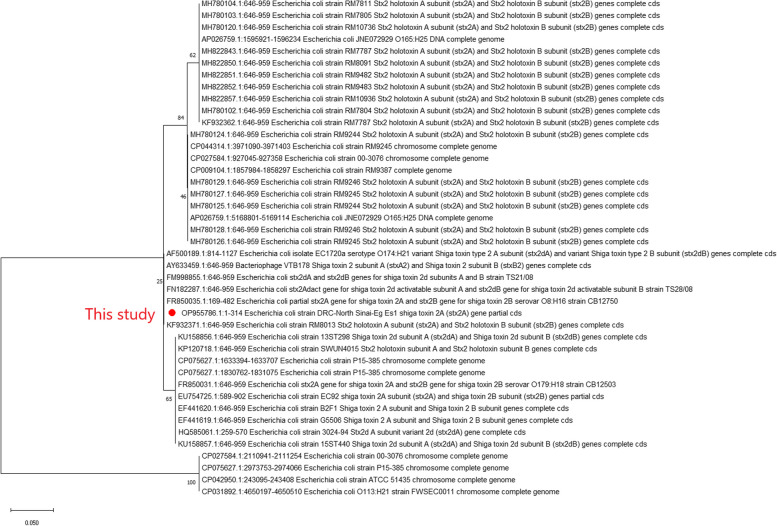
Phylogenetic tree of the OP955786 E. coli strain DRC-North Sinai-Eg Es1 Shiga toxin 2A (Stx2A ) partial sequence. The tree with the highest log likelihood (-204.41) is shown. This analysis involved 42 nucleotide sequences. There were a total of 98 positions in the final dataset

**Fig. 6:**
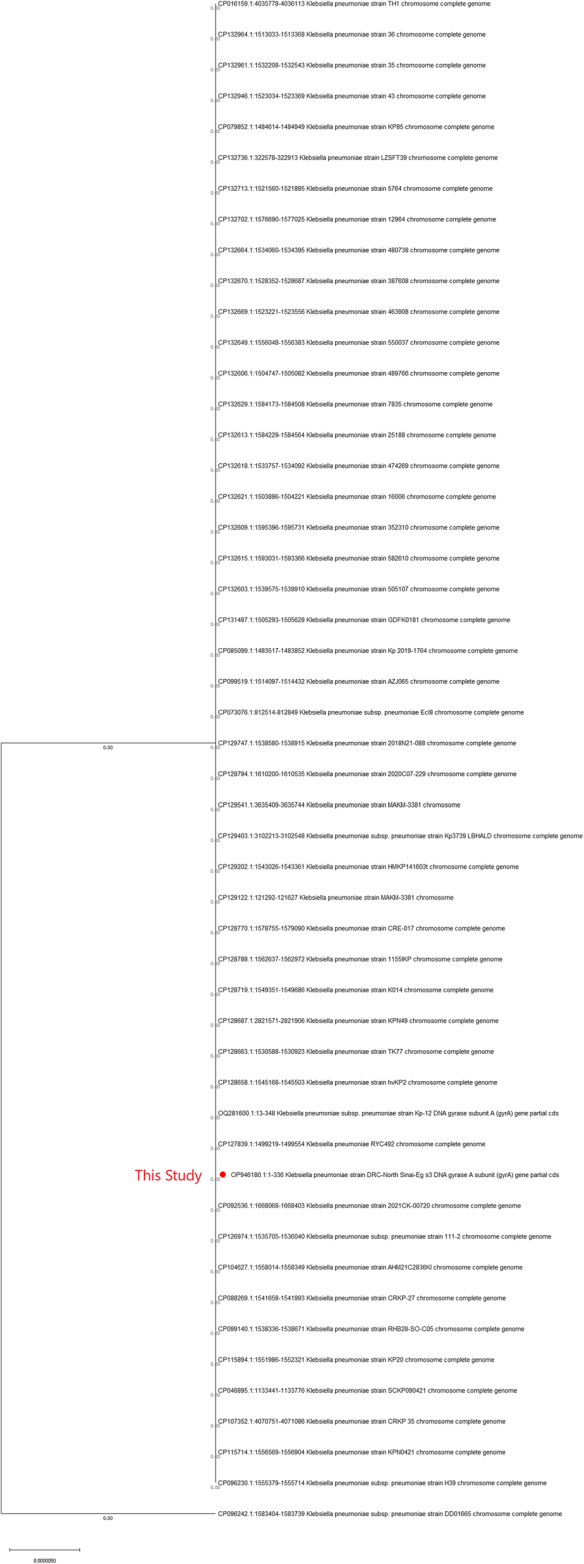
Phylogenetic analysis of the OP946180 *Klebsiella pnuemoniae* strain DRC-North Sinai-Eg s3 DNA gyrase A subunit (gyrA) gene partial sequence. The tree with the highest log likelihood (-461.70) is shown. This analysis involved 50 nucleotide sequences. There was a total of 336 positions in the final dataset

Sequence analysis for *E. coli* strain DRC- North Sinai- Eg was placed in The National Center for Biotechnology Information (NCBI) with accession numbers OP955786 for Stx2A, OP997748, and OP997749 for the Eae gene. For the hylA gene, the accession number was OP946183. *Klebsiella pneumoniae* strain DRC- North Sinai- Eg was OP946180. Evolutionary analysis for OP955786 *E. coli* strain DRC-North Sinai- Eg Figure 5 Strain *E. coli* DRC- North Sinai- Eg Es1 Shiga toxin 2A (Stx2A ) partial sequence . Evolutionary analysis of *Klebsiella pneumoniae *Figure 6 of the OP946180 *Klebsiella pneumoniae* strain DRC- North Sinai- Eg s3 DNA gyrase A subunit (gyrA) gene partial sequence.

### Linkage between antibiotic sensitivity test and antibiotic resistant gene

Ninety-nine percent of *E*. *coli* isolates carried bla^TEM^ genes, and was 100% among *Klebsiella pneumoniae* isolates. At the same time, antibiotic sensitivity tests revealed that 57% of *E*. *coli* isolates and 100% of *Klebsiella pneumoniae* were resistant to amoxicillin. On the other hand, 85% of *Klebsiella pneumoniae* were cefadroxil-resistant, and 78% were ceftazidime-resistant.

## Discussion

In light of the recommendations of the World Health Organization, it is of interest to refer to herbs and natural medicinal plants to address infectious epidemic diseases and common diseases then to attempt to benefit from natural sources, especially desert plants rich in phenolic compounds, which are considered one of the most important pharmaceutical materials. The choice fell on *Moringa oleifera* because of its medical importance in ancient times and its therapeutic effects. It can be cultivated and benefit from the climatic conditions in Egypt to grow it [29]. discussed that most of the antibiotics available today come from natural plants or animals or microbial origin.

In this study, we isolated different pathogens of *Enterobacteriaceae* as a common cause of calf diarrhea. The area of this study was South Sinai, Egypt. It was observed in this study that the secretion of more than one type of *Enterobacteriaceae* along the same calf was increased at younger ages, which was considered a dangerous indicator that might be due to the low immunity of the calf [30]. *E. coli* is a major enteric pathogen known to cause calf diarrhea [31].

We used more than one type of selective and differential media to facilitate the isolation and differentiation process of *Enterobacteriaceae*, and then subsequently performed some biochemical tests for confirmation. MacConkey medium was considered selective, and differential media included crystal violet dye, bile salts, lactose, and neutral red (pH indicator). Crystal violet dye and bile salts inhibit the growth of gram-positive bacteria. Lactose-fermenting *E. coli, and Klebsiella pneumoniae* organic acids producers, principally lactic acid, which lower the pH. Neutral red is a pH indicator that goes colonies pink as the pH drops below 6.8. We used MacConkey sorbitol for screening O157 and completed the molecular identification. MacConkey with sorbitol selected non sorbitol fermenting *E coli:* O157.

Selective media came into being as a result of the identification of antimicrobial agents and their specific targets. These inhibitors enable the removal of undesired bacteria from the microbiota and the selection of desired bacteria [32]. Strains of *E. coli:* O157 do not normally ferment sorbitol, whereas many other serogroups of *E. coli* do ferment sorbitol, and sorbitol MacConkey agar facilitated their isolation [33].

In our study, antibiotic resistance was noted among field isolates by the commercially available discs, and increasing the resistance between the different *E. coli* and *Klebsiella pneumoniae* should be taken into consideration.

The most prominent antibiotics in the treatment of bacterial infections were beta-lactam antibiotics, [34] Penicillins, cephalosporins, cephamycins, and carbapenems were the most famous examples. Beta-lactamase enzyme secretion is considered the main mechanism of bacterial resistance against them [35]. Through the catalysis of amide bond hydrolysis of the four-membered beta-lactam ring [36].

We used microdilution methods directly to detect the MIC for methanol extraction of *Moringa oleifera* based on Eloff’s hypothesis [23] because the complex diffusion of the colored constituent of the extract and some insoluble chemical, highly colored extract hinders or to some extent produces no proper antimicrobial results. The broad ranges of MIC observed after the ELISA reader and normalization of the tested with the optical density of the dilution of methanol extract as a control, positive bacterial control without treatment. MIG ranged from 5 to greater than 50 mg/ ml for *E. coli* and ± 50 mg/ ml for *Klebsiella pneumonae*. Our results prompted us to analyze the methanol extract by HPLC to discover the possible reason for this antimicrobial activity. Ferulic acid was the detected phenolic compound with a concentration of 29,832 ppm.

Ferulic acid mode of action prediction was informed in this study through its stabilization inside the active pocket site of bacterial tyrosinase because of Pi-alkyl, Pi-anion interactions and 4 carbon-hydrogen bonds furthermore, copper ion interactions with certain amino acids revealed a professional suppression effect. The Docking study of ferulic acid with bacterial tyrosinase had not been discussed before. Our in vitro and in silico results support bacterial tyrosinase inhibition.

Molecular docking is a computational technique that expects the binding affinity of ligand molecules to receptor proteins [37]. It has a well-known use in drug discovery and virtual screening of medicines [38, 39].

Tyrosinase is a member of the type-3 copper protein family [40]. It belongs to a group of enzymes, called polyphenol oxidase enzymes in which copper is a constituent in its active site. Two copper atoms are coordinated by conserved three histidine residues [41]. It works only in the oxygenated air to be a dioxygen transporter and then oxidation [42]. As a result, it is responsible for the undesired brown effect of fruits because of oxidase and, melanin formation. Therefore, anti-tyrosinase utilization is common in the food industry. Polyphenolic compounds such as ferulic, caffeic and cinnamic acid have been extensively used as food preservatives [43, 44].

Some microorganisms secrete tyrosinase and then melanin production, it was found that melanin protects bacteria from UV hence, increases its biomass [45] through absorbance of radiation [46]. Melanin also acts as a chelating agent to allow some pathogens to survive under environmental stress [47], it antagonizes the antibiotic through neutralization mechanism and so increasing antibiotic inhibitory dose [48]. Unfortunately, melanin increases the virulence of pathogenic bacteria [46]. A reported review discussed some tyrosinase inhibitors from natural herbal plant extracts, or pure compound derivatives as a potential antibacterial agent [49].

Our report about antimicrobial activity against *E. coli* and *Klebsiella pneumoniae* due to a methanol extract containing ferulic acid is congruent with different researchs [50, 51]. The role of ferulic acid detected in many studies due to its antioxidant scavenger actions and antimicrobial potential [52]. Our results agreed with different studies [53] that mentioned that *E. coli* was more susceptible to ferulic acid with an MIC of 100 mg/ml.

We were interested in studying the virulence and drug resistance genes after this study because they are known to be dangerous for the health of calves experiencing diarrhea as well as for possible transfer to humans, soil, and water. As examples of *Enterobacteriaceae*, *E. coli* and *Klebsiella pneumoniae* pose the greatest threat due to their possession of well-known virulence genes and antibiotic resistance genes, particularly beta-lactam rings.

The bla^CTX−M^ variety of *E. coli* is extensively documented and is rapidly disseminated around the world [54, 55].

African countries have reported a significant incidence of ESBL-producing *E. coli* among humans and animals. The most frequent lactamases found in *Enterobacteriaceae*, particularly in *Escherichia coli* and *Klebsiella pneumoniae*, were SHV, TEM, and CTX-M variants [56].

Although the pathogenicity of *E. coli:* O157 is related to several virulence aspects, in our selected *E. coli*: O157 isolates, fluctuation in virulent gene distribution was observed as only stx1 or stx2 along the same isolates, although they carried both bla^TEM^ and bla ^SHV^. Therefore, the study of antibiotic resistance genes should be performed alongside virulent gene screening. These findings have coincided with similar studies that investigated the occurrence of multiple virulence genes in the same *E. coli:* O157 strain [57].

The increased percentages of virulent STEC *E. coli* in this study, although they ferment the sorbitol on MacConkey sorbitol, drew our attention to these types, and they should be taken into consideration for more research and diagnosis in different laboratories. It can be more dangerous than *E. col*: O157. These findings agreed with those of Oporto et al. (2008) [58] who mentioned that non-O157 STEC is recognized as a significant pathogen with growing effects on human health. *Escherichia coli,* which produces Shiga toxin, has been isolated from a wide range of species, especially ruminants, and cattle thought to be the principal reservoir in developing nations.

To the best of our knowledge, this is the first report concerning bacterial calf diarrhea in desert areas in Egypt, generally and especially in North Sinai. Calves may be seriously endangered by virulent MDR, and ESBL secreted by *E. coli* and *Klebsiella pneumoniae* during diarrhea. Now a known reality requires discussion and solutions that take into account the current state of climate change and sustainable development. To eradicate drug-resistant bacteria naturally, in upcoming visions, desert plants could be a natural source of medical therapeutic solutions.

Virulent-MDR-ESBL *E. coli* and *Klebsiella pneumoniae* prevailed in Calves diarrhea under desert conditions of North Sinai. This study suggests that natural plants may have two potential applications as animal feed additives or as medications for treating zoonotic pathogens. It might be a solution for some zoonotic diseases resulting from current climate changes. It might provide new insight into effective pharmaceutical agents for an animal feed additive to prevent pathogenic bacteria. Attention should be given to newly reclaimed areas regarding health care and limiting the spread of infectious and zoonotic diseases.

### Supplementary Information

Supplementary Material 1.

## Data Availability

The datasets used and/or analyzed during the current study are available at (https://blast.ncbi.nlm.nih.gov/Blast.cgi) with accession numbers OP955786, 0P997748, OP997749, OP946183 and OP946180.

## Abbreviations

MDR- ESBL: Multi Drug Resistant- Extended Spectrum Beta Lactamase
MIC- MBC: Minimum Inhibition Concentration- Minimum Bactericidal Concentration
ppm: Parts per million
V/cm: Volt per centimeter
STEC: Shiga Toxin-producing Escherichia coli
NCD: Neonatal calf diarrhea
EPEC: Entero-Pathogenic Escherichia coli
EHEC: Entero-Hemorrhagic Escherichia coli
STa: Stable toxin
ETEC: Entero-Toxigenic Escherichia coli
Stx 1–2: Shiga toxins 1–2
EHEC hlyA: Entero-Hemorrhagic Escherichia coli hemolysin
HC: Hemorrhagic colitis
SLTI-SLTII: Shiga‐like toxin I-II
hlyA: Hemolysine enzyme encoding gene
N^o^, d, M: Numbers- day- Month
Eae: Adhesion to host cell encoding gene
gyrA: Gene encoding the DNA gyrase A subunit in Klebsiella pneumoniae
WHO: The World Health Organization
HPLC, h: High-performance liquid chromatography, hours
UV, MMFF: Ultra Violet, Merck molecular force field
Nm- m- mm: Nanometer- Millipore- millimeter
mcg/Disc: Microgram/Disc
S- I- R.: Susceptibility- Intermediate- Resistance
®: Positive extended spectrum Beta-lactamase
mg/ml, µg/ml: Milligram/milliliter, microgram/milliliter
DMSO: Dimethylsulfoxide
ELISA: Enzyme Linked Immunosorbent Assay
DNA- PCR: Deoxyribonucleic acid- Polymerase Chain Reaction
F- R: Forward- Reverse
BLAST: Basic Local Alignment Search Tool
ANOVA: Analysis Of Variance
P value- F crit: Probability Value- F critical
TSI- VP: Triple sugar iron- Voges Proskauer
PDB- Å: The protein Data code- Angstroms
Pro: Proline
Arg: Arginine
Glu: Glutamine
Gly: Glycine
His: Histidine
Val: Valine
DG- RMSD: Binding energy- Root-Mean-Square Deviation
kcal/mole- H.B: Kilocalorie/mole- Hydrogen bonds
NCBI: The National Center for Biotechnology Information
