# Supplementary material for: Virulent-MDR-ESBL E. coli and Klebsiella pneumoniae report from North Sinai calves diarrhea and in vitro antimicrobial by Moringa oleifera
Source: BMC Vet Res. 2024 Jun 14;20:259. doi: 10.1186/s12917-024-04088-7 (PMC11179377; doi:10.1186/s12917-024-04088-7)
Supplement: Supplementary file 1 — Supplementary Material 1. [file 12917_2024_4088_MOESM1_ESM.docx]

**Virulent-MDR-ESBL *E. coli and Klebsiella pneumoniae* report from North Sinai calves diarrhea and *In Vitro* antimicrobial by *Moringa oleifera***

***Sahar A. Allam^1, 2^,** **Sara M. Elnomrosy^3^ Samy M. Mohamed^4^**

*1- Infectious Disease Unit, Animal and poultry Health Department, Animal and Poultry Production Division, Desert Research Center*,* 1 Mataria Museum Street, Cairo 11753, Egypt.

*2- Technology Incubator for Nano Agricultural Application, Desert Research Center, Cairo, Egypt.

3- Genome Research Unit, Animal Health Research Institute, Agriculture Research Center, Giza, Egypt.

4- Medicinal and Aromatic Plants Research Department, Pharmaceutical and Drug Industries Research Institute, National Research Center- Al-Buhouth Street, Dokki, Giza, Egypt.

***Corresponding author:** Sahar A*.* Allam [*Saharallam@drc.gov.eg*](mailto:Saharallam@drc.gov.eg)

Table 1S. Antibiotic discs used under study

| **Sympole** | **Commercial name** | **Concentration** | **Family** | **Mode of action( Lorian , 1999)** [**^12^**](../Acceptance%20الحمد%20لله%20والشكر%20لله%20وحده%20لا%20شريك%20له/300dpi/converted/4-marked%20up300dpi%20Sahar%20Allam%20Manusccript-%20Virulent-MDR-ESBL-Moringa-%20%20l.docx%20finalrev%20.docx#Lorian) |
| --- | --- | --- | --- | --- |
| **F** | Nitrofurantoin | **300 mcg/Disc** | nitrofuran family | inhibit DNA synthesis |
| **CN** | Gentamycin | **10 mcg/Disc** | Aminoglycoside | protein synthesis inhibitor |
| **LE** | Levofloxacine | **5mcg/Disc** | fluoroquinolones | Interfere with DNA replication by preventing bacterial DNA from unwinding and duplicating |
| **AK** | Amikacin | **30 mcg/Disc** | Aminoglycoside | protein synthesis inhibitor |
| **E** | Erythromycin | **15 mcg/Disc** | Macrolides | protein synthesis inhibitor |
| **CFR** | Cefadroxil | **30 mcg/Disc** | first-generation cephalosporin –Beta-lactam | Cell-wall  biosynthesis |
| **AZM** | Azithromycin | **15 mcg/Disc)** | Macrolides | protein synthesis inhibitor |
| **DO** | Doxycycline | **30 mcg/Disc** | Tetracyclines | protein synthesis inhibitor |
| **AX** | Amoxicillin | **25 mcg/Disc** | Beta-lactam | Cell-wall  biosynthesis inhibition |
| **CAZ** | Ceftazidime | **30 mcg/Disc** | third-generation cephalosporin | Cell-wall  biosynthesis inhibition |
| **NOR** | Norfloxacine-Fluoroquinolone | **10 mcg/Disc** | fluoroquinolones | Interfere with DNA replication by preventing bacterial DNA from unwinding and duplicating |


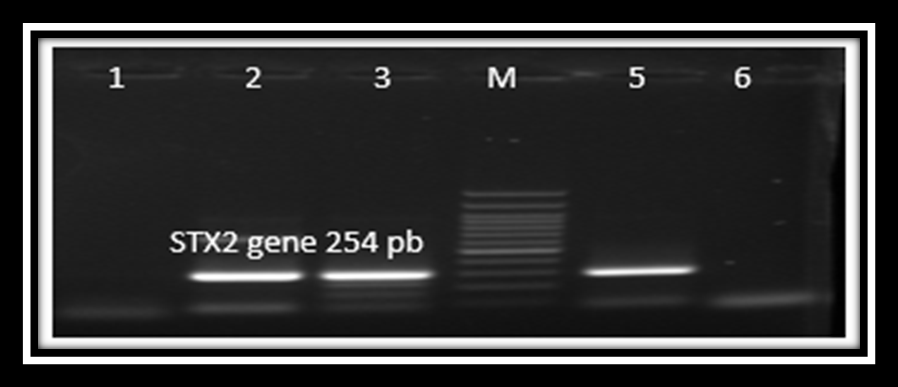


Figure 1S. Agar gel electrophoresis showed results of conventional PCR virulent gene STX2 genes in *E. coli* isolates number (2-3-5) were positive STX2 254bp, 1and 6 were negative. M: represented the molecular size marker (100pb ladder)


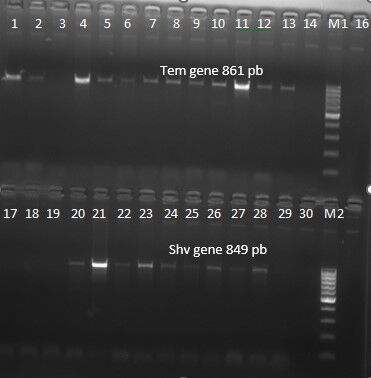


17 18 19 20 21 22 23 24 25 26 27 28 29 30 M2

Figure 2S. Agar gel electrophoresis showed results of conventional PCR Antibiotic resistance of bla^TEM^ gene of *E. coli* isolates number (1-2,4-13) were positive bla^TEM^ gene 861bp. Lane 3 and 14 were negative. M1: represented the molecular size marker (100pb ladder). Isolates number (20-21-22-23-24-25-26-28) were positive to of bla^Shv^ gene 849bp of *E. coli* isolates. Lane 17, 18, 19 and 29 were negative. M2: represented the molecular size marker (100pb ladder)


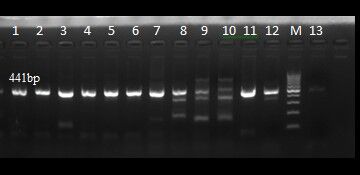


Figure 3S. Agar gel electrophoresis showed results of conventional PCR of virulent gyrA- gene of *Klebsiella* *pneumoniae* number (1-13) were positive gyrA-gene 441bp. M: represented the molecular size marker (100pb ladder)


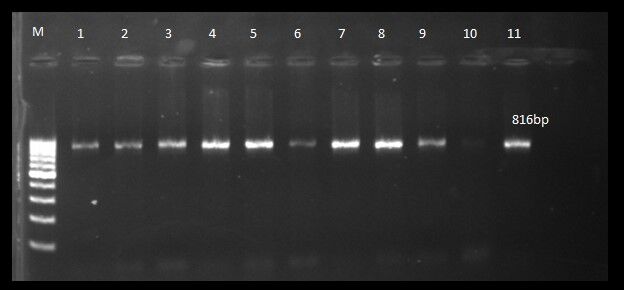


816bp

Figure 4S. Agar gel electrophoresis showed results of conventional PCR antibiotic resistance of bla^TEM^ gene of *Klebsiella* *pneumoniae* number (1-11) were positive bla^TEM^ 816bp. M: represented the molecular size marker (100pb ladder)
